# Intraspecific diversity in *Sinningia speciosa* (Gesneriaceae: Sinningieae), and possible origins of the cultivated florist's gloxinia

**DOI:** 10.1093/aobpla/pls039

**Published:** 2012-11-30

**Authors:** David Zaitlin

**Affiliations:** Kentucky Tobacco Research & Development Center, University of Kentucky,1401 University Drive, Lexington, KY 40546, USA

## Abstract

**Background and aims:**

The florist's gloxinia is a familiar houseplant in the Gesneriaceae, the botanical family that includes the African violet (*Saintpaulia*) and other ornamental species. The gloxinia's wild progenitor is *Sinningia speciosa* (Lodd.) Hiern, a Brazilian endemic. Although it has been cultivated for almost 200 years, little is known about the genetic diversity in *S. speciosa*, how the wild populations relate to one another or even where the cultivated forms originated. Using available wild collections, preliminary phenetic and phylogenetic investigations were conducted to elucidate the interspecific relationships within *S. speciosa* and to infer the origins of the cultivars.

**Methodology:**

Amplified fragment length polymorphism (AFLP) analysis was applied to 24 accessions of *S. speciosa* (17 wild collections, seven cultivars) and one accession each of *Sinningia guttata* and *Sinningia macrophylla*. A maximum likelihood (ML) tree was also calculated from an alignment of the nuclear ribosomal internal transcribed spacer sequence from the same 26 accessions.

**Principal results:**

Dice/UPGMA and principal coordinates analysis of the AFLP data partitioned *S. speciosa* into several distinct clusters, one of which included *S. macrophylla*. All cultivated ‘gloxinias’ grouped together in a major cluster with plants from Rio de Janeiro. The AFLP results were compared with a phylogenetic analysis of the ribosomal spacer region, which was informative in *S. speciosa*. The ML tree generally supported the AFLP results, although several clades lacked strong statistical support.

**Conclusions:**

Independent analyses of two different data sets show that *S. speciosa* is a diverse species comprised of several lineages. Genetic distance estimates calculated from the AFLP data were positively correlated with geographic distances between populations, indicating that reproductive isolation could be driving speciation in this taxon. Molecular markers are under development for population genetic studies in *S. speciosa*, which will make it possible to define evolutionarily significant units for purposes of conservation.

## Abstract (precis)

This research examines the relationships between 24 accessions (17 wild, 7 cultivars) of *Sinningia speciosa*, the florist’s gloxinia. Phenetic and phylogenetic methods together suggest that distinct geographic lineages exist within this taxon, which will be important for future conservation efforts.

## Introduction

**Fig. 1:**
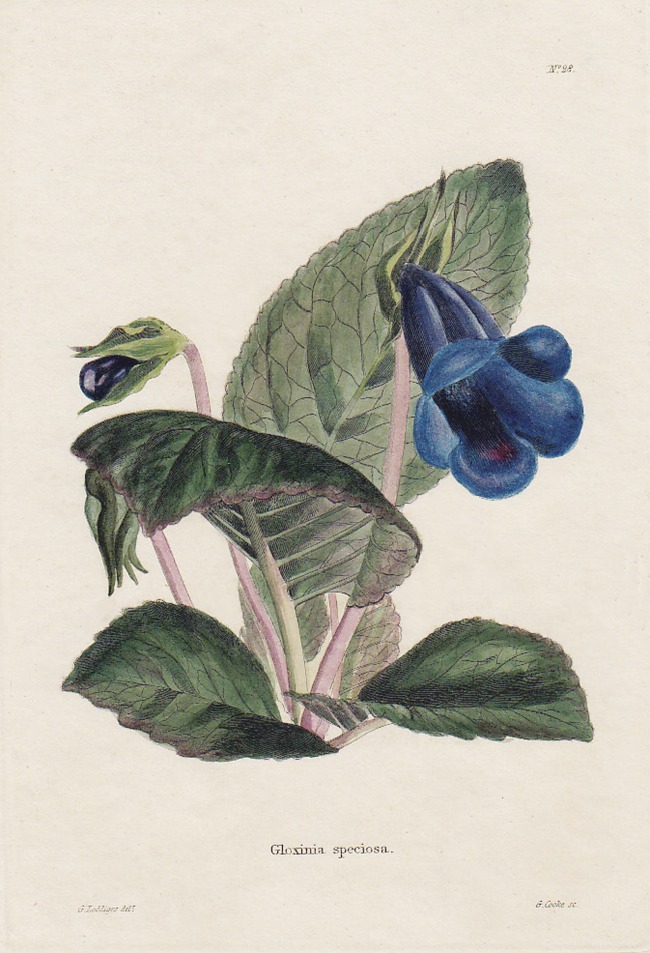
**The first known published image of *Gloxinia* (*Sinningia*) *speciosa* from Volume I (third fascicle) of *The Botanical Cabinet*, plate number 28, July 1817.** Illustration by George Loddiges (son of Conrad Loddiges), copper-plate engraving by George Cooke.

*Sinningia speciosa* is a herbaceous, tuber-forming perennial native to south-eastern Brazil, and is one of the ∼70 species in the genus *Sinningia* (Gesneriaceae, tribe Sinningieae) (Chautems *et al*. 2010). *Sinningia speciosa* was originally introduced into cultivation in Great Britain in 1815 (Don 1838; Harrison 1847). The first known collection was made by Allan Cunningham (1791–1839) and his partner James Bowie (1789–1869), both of whom were employed as Botanical Collectors in Brazil by the Royal Botanic Gardens, Kew, from 1814 to 1816 (Heward 1842; Britten and Boulger 1893; Smith 1911). Joachim Conrad Loddiges (1738–1826), the owner of a large and prestigious nursery in the village of Hackney (now a borough of London), introduced *S. speciosa* to the public with an illustration in the first issue of *The Botanical Cabinet* (Loddiges 1817), published by his son George from 1817 to 1833 (Fig. 1). Loddiges named the new plant *Gloxinia speciosa*, placing it in *Gloxinia* L'Héritier, an existing genus of rhizomatous perennial herbs in the Gesneriaceae from Central and South America. The flowers of the type species, *Gloxinia perennis*, are outwardly similar to those of *S. speciosa*, and Loddiges' understandable error accounts for the common name still in use today (*Sinningia* Nees was not erected until 1825, but he did choose the correct family). A copy of the *Catalogue of Plants Which are Sold by Conrad Loddiges and Sons, Nurserymen, at Hackney, Near London* (12th ed.) has survived in the library of the Arnold Arboretum of Harvard University. From this, we know that the nursery was offering both *G. maculata* (=*G. perennis*) and *G.* (*S.*) *speciosa* for sale by 1820. The new ‘gloxinia’ was eagerly sought by plant collectors and was soon ‘ … to be found in most of the large collections about town [London]’ (Sims 1817).

The first botanical descriptions of *S. speciosa* (as *G. speciosa*) were published in two British botanical periodicals in 1817; one was by John Sims, MD in *Curtis's Botanical Magazine* (Sims 1817), and the other, in both Latin and English, is from *The Botanical Register* and is attributed to John B. Ker Gawler (Edwards 1817). The designated type specimen for *S. speciosa* is held in the collection of the Conservatoire et Jardin Botanique de la Ville Genève (G) as specimen G00133692; the herbarium sheet is dated 23 June 1819, and the notation states that the plant was obtained from ‘jard. de Loddiges a Hackney’ (**www.ville-ge.ch/musinfo/bd/cjb/chg/adetail.php?id=106139&base=img&lang=en**). Modern descriptions of *S. speciosa* can be found in Bailey (1917) and Moore (1957), although neither mentions the perennial tuber.

**Fig. 2:**
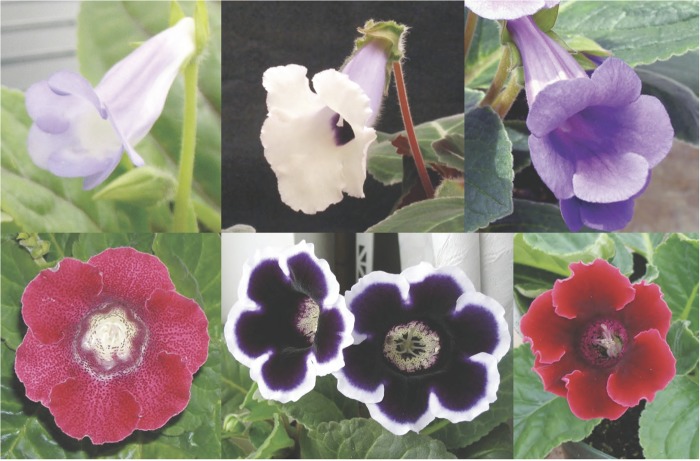
**Floral diversity in *S. speciosa***. The top row shows flowers of three wild forms: ‘Búzios’ from the Cabo Frio region of south-eastern Rio de Janeiro state (left), ‘Pedra Lisa’ from northern Rio de Janeiro state (centre) and ‘São Conrado’ from the southern part of Rio de Janeiro city. The bottom row shows three cultivars with large peloric flowers in colours and patterns unknown in the wild.

Even though *S. speciosa* was formally transferred to *Sinningia* in 1877 (Hiern 1877), the original name *G. speciosa* remained in use for many years, and is occasionally encountered in the scientific literature from the 20th century (e.g. Bushee 1908). This confusion is understandable because of the many discarded synonyms for *S. speciosa* (see below), and also because the common name ‘gloxinia’ came into general use for this species. Improved cultivars have been grown as ornamental houseplants and in greenhouses for over 160 years in Great Britain, continental Europe and the United States, and are still grown to this day. Wild forms of *S. speciosa* all have nodding, bilaterally symmetrical flowers that are lavender or purple (rarely white or pink) in colour. Cultivated varieties, however, have fully erect, radially symmetrical (peloric) flowers in colours ranging from white to purple and red, and often with unusual corolla patterning (Fig. 2). Of the many hundreds of cultivars named and sold in the 19th and early 20th centuries, few have survived to the present day; examples of some that are still available are ‘Emperor Fredrick’, ‘Emperor William’ (aka ‘Kaiser Wilhelm’), ‘Defiance’ and ‘Blanche de Meru’.

The distribution of *S. speciosa* is restricted to the Atlantic coastal forests (Mata Atlântica) of south-eastern Brazil (Araujo and Chautems 2010), an area recognized as one of 34 world biodiversity hotspots because of its high level of species richness and endemism (Myers *et al*. 2000; Mittermeier *et al*. 2004). The geographical range of *S. speciosa* encompasses ∼4 × 10^5^ km^2^ in Espírito Santo, Minas Gerais and Rio de Janeiro states, and populations may also exist in São Paulo, Paraná and Santa Catarina states (A. Chautems, Conservatoire et Jardin Botaniques de la Ville de Genève, personal communication). Human activities such as agriculture (cattle ranching; sugarcane, eucalyptus and coffee plantations), mining, and expansion of the major cities of Rio de Janeiro and São Paulo have greatly reduced the size of the southern coastal forests, threatening the genetic diversity of the plant and animal species that live there. Despite this loss of habitat, many natural populations of *S. speciosa* survive, and the species as a whole has retained considerable morphological diversity (Zaitlin and Pierce 2010). Variation is readily observed in the leaves (size, shape, colour, indument), flowers (size, shape, number per axil), corolla (colour, shape, patterning), petal lobes (size, shape), calyx (size, shape) and stem (colour, internode length) [see Additional Information]. Past taxonomic treatments of *S. speciosa* have left us with a list of discarded synonyms for this diverse species encompassing 39 taxa in seven genera that included *Ligeria*, *Orthanthe*, *Orobanche* (Skog and Boggan 2007) and *Martynia* (Loiseleur-Deslongchamps 1820) in addition to *Gloxina* and *Sinningia*.

The objective of the research presented here was to determine whether amplified fragment length polymorphisms (AFLPs) are informative in *S. speciosa*, and to test the hypothesis that they can be used for investigating genetic diversity within a group of wild and cultivated accessions. While the main goal of this preliminary study was to define the intraspecific relationships between plants collected from natural populations, seven domesticated cultivars were also included to shed some light on the geographical origins of the cultivated florist's gloxinia. Amplified fragment length polymorphism is a powerful and robust molecular marker technology that requires no prior knowledge of genome sequence, and is therefore potentially useful in non-model and genetically uncharacterized organisms. Amplified fragment length polymorphisms have been widely applied in studies of plant genetic diversity and evolution (Sudupak *et al*. 2004; Spooner *et al*. 2005; Zerega *et al*. 2005; Allen *et al*. 2008; Azizi *et al*. 2009; Garrido *et al*. 2012), genotyping and cultivar identification in ornamental and horticultural crops (Chao *et al*. 2005; Parks *et al*. 2006; Krichen *et al*. 2008; Wu *et al*. 2011), and as an aid in resolving species-level phylogenies when DNA sequence diversity is limited (Beardsley *et al*. 2003; Després *et al*. 2003; Pelser *et al*. 2003; Mereďa *et al*. 2008). In the present study, an analysis of fluorescent end-labelled AFLP fragments amplified from genomic DNA was used to infer genetic relationships within a group of *S. speciosa* accessions. Included were 15 wild collections of *S. speciosa* of known origin, two wild-type collections of unknown origin, seven ‘gloxinia’ cultivars and two related species (*Sinningia guttata* and *Sinningia macrophylla*) as potential outgroups. The 26 plant accessions were clustered using phenetic (Dice/UPGMA) and principal coordinates analyses (PCoA) of the AFLP data. These results were compared with a phylogenetic analysis of the nuclear ribosomal internal transcribed spacer (nrITS) region amplified and sequenced from the same 26 *Sinningia* taxa. Taken together, the two independent analyses show that *S. speciosa* is a highly diverse taxon that may include *S. macrophylla*, and that the *S. speciosa* cultivars appear to be most closely affiliated with plants collected from populations in or near the city of Rio de Janeiro.

## Methods

### Plants and DNA

The majority of plants used in this study were grown from seeds, with several exceptions as detailed below. Eight of the wild-collected *S. speciosa* accessions, as well as the cultivars ‘Guatapara’ and ‘Dona Lourdes’, were obtained as seeds from M. Peixoto (Mogi das Cruzes, SP, Brazil) and are described in Zaitlin and Pierce (2010). The wild *S. speciosa* collections ‘Massaguassu’ (from near a beach of that name in the municipality of Caraguatatuba in SP state) and ‘CM’ (unknown origin, possibly from Rio de Janeiro), and the cultivar ‘Purple’ were also obtained from M. Peixoto. Wild-type *S. speciosa* accessions AC1503, ‘Chiltern Seeds’ and ‘Regina’, and the peloric cultivar ‘Red with Spots’ were purchased from the seed fund of the Gesneriad Society (**www.gesneriadsociety.org**). Seeds of the peloric white-flowered cultivar ‘Ken's White’ were obtained from the Gesneriad Hybridizer's Association. ‘Avenida Niemeyer’ was the kind gift of Wallace Wells of New York, NY; the original collection was made in August 1975 by Charles Marden Fitch on a rocky slope between the Sheraton Rio Hotel and the Hotel Nacional in Rio de Janeiro on the coastal road of this name. *Sinningia speciosa* ‘São Conrado’ was collected in the same general area in 1999, several kilometres to the west, by Mr Tsuh Yang Chen of New York City, and was the gift of Dr William Price of Vancouver, BC, Canada. ‘Kaiser Wilhelm’, a classic ‘gloxinia’ cultivar with peloric purple and white flowers, was obtained as a tuber from a commercial source. DNA of JFS4512, a peloric cultivar, was kindly provided by Dr James Smith of Boise State University (Smith *et al*. 2004). *Sinningia* seeds were routinely germinated in 10 cm pots containing Pro-Mix BX (Premier Horticulture Inc., Quakertown, PA, USA), and the seedlings were grown under fluorescent lighting (80 µmol m^−2^ s^−1^) at 25 °C until they were large enough to sample for DNA extraction.

Total plant DNA was extracted from small samples of leaf tissue (pooled from several plants of each accession) using the DNeasy Plant Mini Kit (Qiagen Inc., Valencia, CA, USA). DNA concentration was determined by absorbance at 260 nm using a Nanodrop ND-1000 spectrophotometer (Thermo Fisher Scientific, Wilmington, DE, USA).

### AFLP methods

Amplified fragment length polymorphism manipulations were performed essentially as described by Vos *et al*. (1995), using a modification that allowed for fluorescent detection of the amplified fragments. An AFLP Core Reagent Kit (catalogue #10482-016) was purchased from Invitrogen (Carlsbad, CA, USA). Fluorescent Eco + 3 primers were 5′-end labelled with the WellRed D4 dye (Beckman Coulter, Fullerton, CA, USA), and were synthesized by Integrated DNA Technologies, Inc. (IDT: Coralville, IA, USA). Unlabelled primers were also purchased from IDT. Genomic DNA samples (50–100 ng) were digested to completion with EcoRI and MseI at 37 °C in a final volume of 25 µL. Following ligation of the EcoRI- and MseI-specific adaptor sequences for 15 h at 20 °C, the reactions were diluted 10-fold into 10 mM Tris-HCl, 0.1 mM EDTA (pH 8.0). Pre-selective amplifications (PSA) and selective amplifications (SA) were performed exactly as described by Zhang *et al*. (2012). A C1000 Thermal Cycler (Bio-Rad Laboratories, Hercules, CA, USA) was used for all AFLP amplifications. Selective amplification reactions were diluted 30-fold into sample loading solution (Beckman Coulter) containing a 1/100 dilution of DNA Size Standard-600 (Beckman Coulter) for analysis. DNA fragments were separated by capillary electrophoresis on an automated DNA sequencing instrument (Beckman Coulter CEQ8000 Genetic Analysis System) using the Frag-4 method (capillary temperature = 50 °C, denaturation 90 °C for 2 min, sample injection 30 s at 2 kV, electrophoretic separation 65 min at 4.8 kV). Sequences of the AFLP primers used are given in Table 1.

**Table 1:** **DNA sequences of oligonucleotide primers used for AFLP analysis and amplification and sequencing of the nrITS region in *Sinningia*.** Amplified fragment length polymorphism primers E32 (Eco + AAC) and E33 (Eco + AAG) were labelled at the 5′ termini with WellRed dye D2.

**Primer name**	**DNA sequence (5′→3′)**
E01	AGACTGCGTACCAATTCA
M02	GATGAGTCCTGAGTAAC
E32	GACTGCGTACCAATTCAAC
E33	GACTGCGTACCAATTCAAG
M48	GATGAGTCCTGAGTAACAC
M49	GATGAGTCCTGAGTAACAG
M50	GATGAGTCCTGAGTAACAT
ITS2Gm	TGACGCCCAGGCAGACGT
ITS3P	GCATCGATGAAGAACGTAGC
ITS5seq2	CAAGGTTTCCGTAGGTGAACCTG
ITS6	GCGAGAAGTCCATTGAACC
ITS8m	GACGCTTCTCCAGACTACA

DNA fragment peak sizes in the D2 channel were calculated against the standards in the D1 channel using the software supplied with the CEQ8000 instrument. The quartic equation gave the best approximation of a linear relationship between peak sizes (in nucleotides) vs. migration time with the 600 standard. Data peaks were machine scored (1 = present, 0 = absent) using the ‘New AFLP Analysis’ module (parameter settings: 10 % slope threshold, 10 % relative peak height threshold, 95 % confidence level, bin width = 1 bp, PA ver. 1 dye mobility calibration) and the data exported to Microsoft Excel. Every AFLP chromatogram was examined for the presence of unscored peaks at a minimum height threshold of 5000 fluorescence units. Data entries from poorly resolved and miscalled peaks were removed from the spreadsheets. Peaks well outside the standard size range (<60 and >640 bases) were also removed. Similarity matrices based on the Dice coefficient (Dice 1945) were calculated from the binary AFLP data using the SIMQUAL module in the NTSYSpc 2.2 software package (Rohlf 2009), and clustering was performed with the UPGMA (unweighted pair-group method with arithmetic mean) algorithm in the SAHN module. Goodness of fit of the UPGMA dendrogram to the Dice similarity matrix was assessed using the COPH and MXCOMP modules in NTSYSpc. Bootstrapping (BS) was also performed with NTSYSpc using the program modules RESAMPLE to generate 1000 resampled files of the AFLP data, SIMQUAL to calculate the similarity matrices and CONSENS (with MAJRUL selected) to compare the trees and calculate cluster frequencies. Principal coordinates analysis was performed on the AFLP data (imported in spreadsheet format) using the statistical software package MVSP 3.2 (Kovach Computing Services, Anglesey, UK) with the following program settings: data matrix transformed, Euclidean distances computed, eigenanalysis tolerance set to 10^−7^. The resulting graphs were exported as enhanced metafiles, and text labels were added with the program Metafile Companion (Companion Software, Sunderland, MA, USA).

Genetic distances (GDs) were calculated with NTSYSpc for the AFLP data (GD = 1−Dice's coefficient) and MEGA 5.03 (Tamura *et al*. 2011) for the nrITS DNA sequence data (Tamura–Nei substitution model), respectively. Geographic distances were calculated with the GPS Visualizer tool (**http://www.gpsvisualizer.com/calculators**) based on GPS coordinates for Brazilian cities and towns kindly provided by A. Chautems. *Sinningia speciosa* ‘Jurapê’ was named for the mountain in Santa Catarina state where it was allegedly collected, and coordinates for the nearest town (Joinville) are S26°18′16.2″, W48°50′55.248″ (−26.3045, −48.84868). Serra da Vista is a mountain ∼20 km south of the town of Cardoso Moreira in Rio de Janeiro state. Coordinates used for this collection were S21°39′55.116″, W41°36′59.4″ (−21.66531, −41.6165). Mantel tests were performed with IBDWS (Isolation-by-Distance Web Service v.3.23; Jensen *et al*. 2005) to determine whether the GD matrix was significantly correlated with the geographic distance matrix. Ten thousand randomizations were used.

### Nuclear ribosomal internal transcribed spacer region amplification and DNA sequencing

The internal transcribed spacer region of the 18S–26S nuclear rDNA repeat (nrITS) was amplified from *Sinningia* genomic DNA (10–20 ng) using primers ITS6 (this study; see Table 1 for primer sequences) and ITS8m (modified from Möller and Cronk 1997) in 1× FailSafe ‘A’ premix (Epicentre Biotechnologies, Madison, WI, USA) at a concentration of 1 µM in the polymerase chain reaction (PCR; 30 cycles of 95 °C for 30 s, 56 °C for 60 s and 72 °C for 90 s, followed by one cycle of 72 °C for 5 min). ITS6 and ITS8m are complementary to conserved sequences at the 3′-end of the 18S rRNA gene and the 5′-end of the 26S rRNA gene (see fig. 1 of Baldwin *et al*. 1995) from *Sinningia*, respectively. All nrITS gene fragments (∼850 bp) were purified using the QIAquick PCR Purification Kit (Qiagen) to remove salts and unincorporated primers. Nucleotide sequencing was performed on an automated sequencing instrument (Beckman Coulter CEQ8000) with four passes (two per strand), using primers ITS5seq2 and ITS8m, both of which direct DNA strand synthesis from the fragment ends, and ITS2Gm and ITS3P, which are complementary to the ends of the 5.8S rDNA sequence, but are of opposite polarities (Möller and Cronk 1997). The nrITS regions from all *Sinningia* accessions reported here were sequenced at least twice, and the sequence tracings were carefully examined to resolve any possible conflicts or errors. The nrITS region from the cultivar ‘Kaiser Wilhelm’ proved to be polymorphic; the amplified fragment was therefore cloned into pGEM-T (Promega, Madison, WI, USA), and the inserts in several recombinant plasmids were sequenced with the same four primers.

Alignment of the nrITS region was performed manually using the program Se-Al v2.0a11 (Rambaut 2007), which allowed the alignment to be exported and saved in FASTA format. Maximum likelihood analysis was performed on the *Sinningia* nrITS alignment with MEGA 5.03 for Mac OS X (Tamura *et al*. 2011), using the Tamura–Nei substitution model with gamma distribution (TN93 + G) to estimate rate variation across nucleotide sites. Reliability of the phylogenetic tree was estimated by BS with 1000 replications.

## Results

### AFLP

**Fig. 3:**
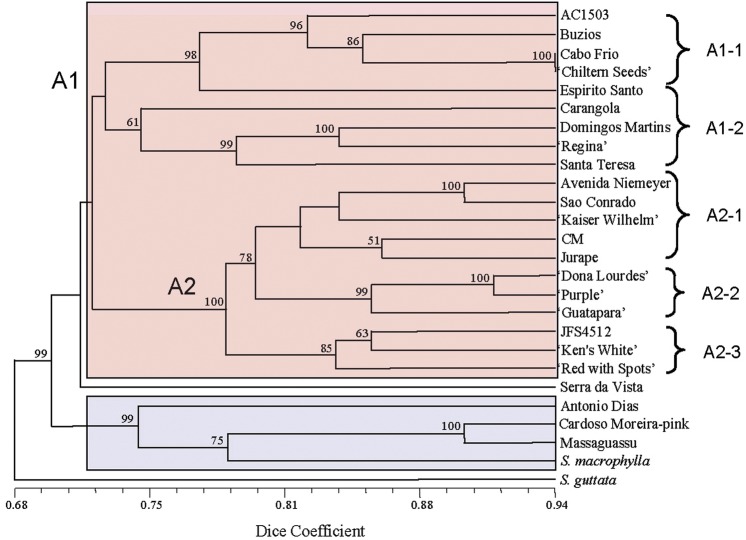
**Dice/UPGMA tree showing phenetic relationships within a group of 24 *S. speciosa* accessions (17 wild and seven cultivars) based on AFLP data**. Two related species, *S. guttata* and *S. macrophylla*, were also included in the analysis. Major clusters A and B are shown in pink and blue, respectively. Secondary clusters A1 and A2 are labelled at the appropriate nodes. Tertiary clusters A1-1, A1-2, A2-1, A2-2 and A2-3 are indicated by brackets. Bootstrap percentages >50 % are shown adjacent to the cluster nodes.

Sixteen AFLP primer pairs were initially screened against a set of six *S. speciosa* genotypes, and four that gave a relatively uniform peak distribution were chosen and were informative in the group of test samples (data not shown). Amplified fragment length polymorphism profiles were generated for the 26 *Sinningia* accessions using the E + 3/M + 3 primer combinations E32M48, E32M50, E33M49 and E33M50. Amplified fragment length polymorphism peaks were scored as 1 = present, 0 = absent. Of the 602 scored fragment bins, 591 (98.2 %) were polymorphic in this sample set. The 11 monomorphic bins were removed, as were four that were outside the range of the size standards. Within the 24 accessions of *S. speciosa* (*S. guttata* and *S. macrophylla* removed) there were 563 bins, of which 558 (99 %) were polymorphic (Table 2). The final data matrix consisted of 15 264 binary scores (26 accessions × 587 bins). A Dice/UPGMA phenogram derived from the AFLP data is shown in Fig. 3. The cophenetic correlation coefficient (normalized Mantel statistic *Z*) was *r* = 0.94651, indicating that the tree was significantly correlated with the similarity matrix from which it was computed. Bootstrap values for all nodes present in the consensus tree ranged from 51 to 100 %, with a mean of 88.8 %. The same analysis performed using Jaccard's coefficient (Jaccard 1908) produced a very similar but more complicated tree with a slightly higher correlation coefficient (*r* = 0.95526) and identical BS percentages for the conserved nodes (not shown).

**Table 2:** **Number, sizes and relative polymorphism of AFLP markers amplified from *Sinningia* genomic DNA with four primer combinations.** In determining the number and per cent of mono- or polymorphic AFLP markers, ‘all *Sinningia*’ includes the wild and cultivated accessions of *S. speciosa* as well as one accession each of *S. guttata* and *S. macrophylla*.

**AFLP**					
Primer pair	E32M48	E32M50	E33M49	E33M50	Totals
No. of bins	115	155	130	202	602
Mean no. of peaks/entry	22.7	41.2	34.6	64.2	
Size range (bases)	60–638	59–552	57–671	60–690	
Number/% monomorphic (all *Sinningia*)	3/2.6	2/1.3	1/0.8	5/2.5	11/1.8
Number/% polymorphic (all *Sinningia*)	112/97.4	153/98.7	129/99.2	197/97.5	591/98.2
Number/% monomorphic (*S. speciosa*)	3/2.8	4/2.6	2/1.6	7/3.6	16/2.8
Number/% polymorphic (*S. speciosa*)	103/97.2	148/97.4	125/98.4	188/96.4	564/97.2

To estimate the level of inherent error in the AFLP procedure, four independent AFLP amplifications were performed on DNA from *S. speciosa* ‘Guatapara’ that had been digested with EcoRI and MseI. Adaptor ligations, PSA reactions with primers E01 and M01, and SA reactions with primer pair E32M50 were carried out as described earlier (Zhang *et al*. 2012). Six replicates of each SA were then separated on the CEQ8000 instrument, and the data analysed as for the experimental samples. The E32M50 primer pair gave a total of 76 fragment bins between 67 and 570 nucleotides. This included three bins with only a single entry (unique peaks), which were removed from the analysis. Pair-wise similarities (simple matching) for the six within-group replicates were 0.945–0.986 (group 1), 0.959–0.973 (group 2), 0.973–1.00 (group 3) and 0.973–1.00 (group 4). This translates into error rates of 0–5.5 %, which is within the range cited by Meudt and Clarke (2007) for other plant AFLP studies. There were 17 peaks that were either present or absent for all entries within one or more replicate sets. Examination of the chromatograms revealed that this was due to minor between-run variances in the instrument scoring, which resulted in the peaks being placed in a new bin ±1 nucleotide of the expected size, and was readily corrected.

Dice/UPGMA analysis of the AFLP data provided evidence of genetic diversity within *S. speciosa*. Two major clusters were evident in the phenetic tree, with 20 of the 26 accessions present in cluster A (although there is a major division within this large cluster) (Fig. 3). The placement of ‘Serra da Vista’ could imply the existence of a third cluster, although its position between clusters A and B makes its affiliations inconclusive. Cluster A1 consisted of three plants collected from Rio de Janeiro state (AC1503, ‘Buzios’ & ‘Cabo Frio’), one plant from the southern part of Minas Gerais (‘Carangola’) and three plants from Espírito Santo state (‘Espirito Santo’, ‘Santa Teresa’ and ‘Domingos Martins’). Cluster A2 contained two plants from Rio de Janeiro (‘Avenida Niemeyer’ and ‘São Conrado’), the unidentified wild accession ‘CM’, and all of the domesticated varieties (‘Kaiser Wilhelm’, ‘Dona Lourdes’, ‘Guatapara’, ‘Purple’, JFS4512, ‘Ken's White’ and ‘Red with Spots’). Also in cluster A are two wild-type accessions of unknown geographical origins in A1 (‘Regina’ and ‘Chiltern Seeds’), and ‘Jurapê’, an unconfirmed collection from Santa Catarina state, in A2. Cluster B contained one collection each from Rio de Janeiro and São Paulo states (‘Cardoso Moreira-pink’ and ‘Massaguassu’, respectively), one from Minas Gerais (‘Antônio Dias’), and *S. macrophylla* from Bahia. *Sinningia guttata* occupied a unique position in this analysis, and was an appropriate outgroup.

**Fig. 4:**
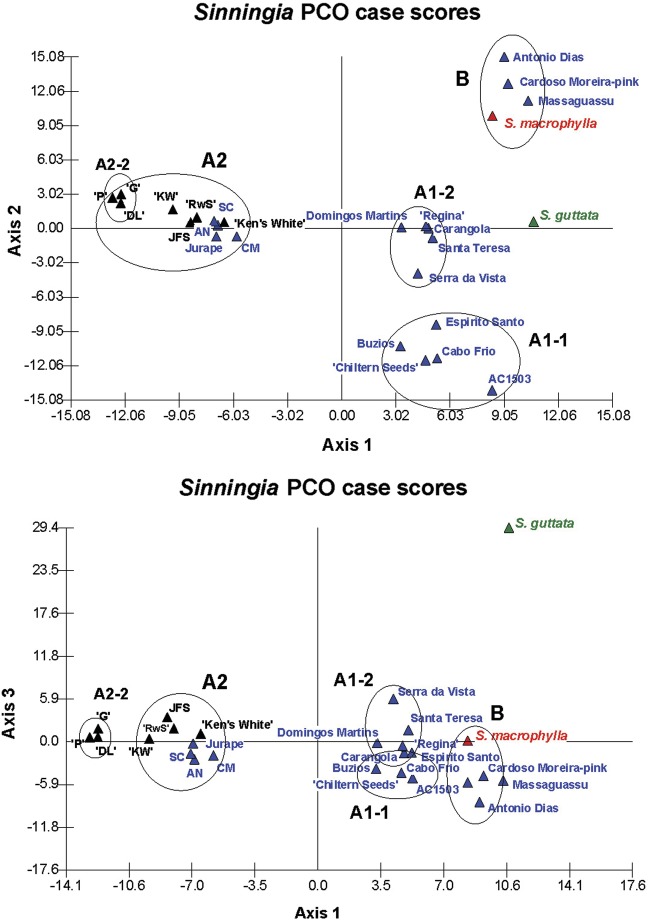
**Principal coordinates analysis of *Sinningia* AFLP data**. The relationships between 24 accessions of *S. speciosa* and single accession each of *S. guttata* and *S. macrophylla* are shown in two-dimensional scatterplots of (A) axes 1 vs. 2 and (B) axes 1 vs. 3. The circled groupings are labelled to show their affiliations with clusters in the Dice/UPGMA tree (Fig. 3). *Sinningia guttata* is shown in green, *S. macrophylla* in red, wild *S. speciosa* are in blue, and the cultivars are in black. Abbreviations used in cluster A2 are: Avenida Niemeyer (AN), São Conrado (SC), ‘Purple’ (‘P’), ‘Dona Lourdes’ (‘DL’), ‘Guatapara’ (‘G’), ‘Kaiser Wilhelm’ ‘(KW’), JFS4512 (JFS) and ‘Red with Spots’ (‘RwS’). Eigenvalues for the first three PCO axes were 11.121, 8.883 and 7.803, respectively.

The AFLP data matrix was also examined by PCoA, a multivariate ordination method used to explore and visualize relationships within a data set. In PCoA, pairwise distances computed between individual variables are projected as coordinates upon a set of derived orthogonal axes, and similar cases grouped together. The distribution of the 26 *Sinningia* accessions is shown in Fig. 4. Eigenvalues for the first three principal coordinates axes (PCO) were 11.121, 8.883 and 7.803, respectively, representing 27.806 % of the total variation. Four well-defined clusters, corresponding to A1-1, A1-2, A2 and B from the Dice/UPGMA phenogram (Fig. 3), were evident in the 2-D scatterplots. *Sinningia guttata* was well separated from the *S. speciosa* clusters and *S. macrophylla* along all three axes (Fig. 4A and B). *Sinningia macrophylla*, which was originally included as a second outgroup species, showed affiliation with the three forms of *S. speciosa* from cluster B—‘Antônio Dias’, ‘Massaguassu’ and ‘Cardoso Moreira-pink’. The placement of ‘Serra da Vista’, which was problematic in the Dice/UPGMA tree, is clearer in the PCO plot, where it was grouped with ‘Carangola’, ‘Domingos Martins’, ‘Santa Teresa’, and ‘Regina’ in subcluster A1-2. Principal coordinates analysis also separated the three cultivars with wild-type flowers (‘Dona Lourdes’, ‘Guatapara’ and ‘Purple) from the peloric cultivars + wild collections in cluster A2. This discrete subcluster was preserved from the Dice/UPGMA tree and is designated A2-2 (Fig. 3).

**Fig. 5:**
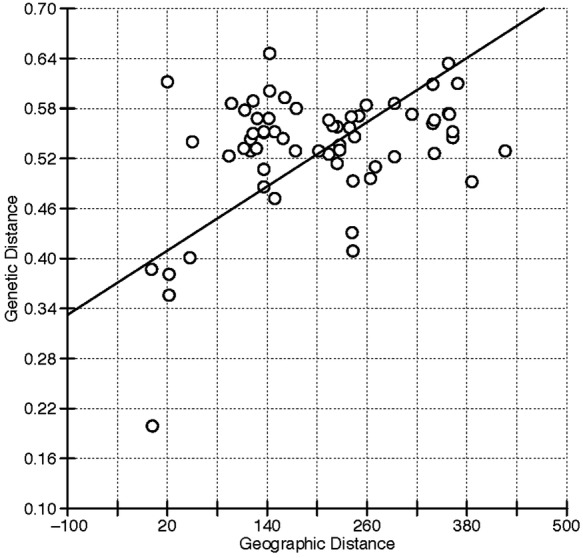
**Isolation-by-distance (IBD) analysis for 12 wild collections of *S. speciosa***. Genetic distances were calculated from the AFLP data using NTSYSpc (Rohlf 2009), and the geographic distances were calculated from GPS coordinates using the web-based GPS Visualizer tool (see the text). The Mantel test of significance and the IBD plot were calculated with IBDWS v. 3.23 (Jensen *et al*. 2005). In the linear regression, the equation for the line was *y* = 0.000642*x* + 0.3966, and *R*^2^ = 0.125. Three unverified *S. speciosa* accessions (‘CM’, ‘Massaguassu’ and ‘Jurapê’) were excluded from this analysis.

Genetic distance estimates derived from the AFLP data were plotted against calculated geographic distances for all pairwise combinations of wild *S. speciosa* accessions. The unverified collections ‘Jurapê’, ‘CM’ and ‘Massaguassu’ were excluded from this analysis. Linear regression of the data shows a positive relationship between genetic and geographic distances for *S. speciosa* in south-eastern Brazil (Fig. 5). The Mantel test, as implemented in IBDWS version 3.23 (Bohonak 2002; Jensen *et al*. 2005), indicated that the matrices of genetic and physical distances were significantly correlated (*Z* = 7525.992, *r* = 0.3530, *P* ≤ 0.0053). This relationship held for all combinations of arithmetic and logarithmic values derived from the AFLP data, but not for GD estimates calculated from phylogenetic comparisons of the nrITS sequences (not shown).

### *Sinningia* nrITS sequences

The full intergenic ITS1-5.8S-ITS2 region was amplified from genomic DNA isolated from the 26 *Sinningia* accessions using primers complementary to conserved sequences at the 3′-end of the 18S and the 5′-end of the 26S rRNA genes (for a map of this region in angiosperms, see Hershkovitz *et al*. 1999). Unambiguous nucleotide sequences were obtained directly from all amplified fragments with the exception of that from *S. speciosa* ‘Kaiser Wilhelm’, which was cloned prior to sequencing. There was no indication of paralogous loci or pseudogene amplification from any accession used in this study (see Baldwin *et al*. 1995; Buckler *et al*. 1997; Álvarez and Wendel 2003; Xiao *et al*. 2010). The criteria used by Besnard *et al*. (2009) suggested that the sequenced nrITS regions are part of functional rDNA repeats (Table 3). In addition, the conserved 14 bp sequence (5′-GAATTGCAGAATCC-3′) that is diagnostic of plant 5.8S RNA genes (Jobes and Thien 1997) was invariant in all *Sinningia* ITS region sequences. Based on comparisons with published ITS sequences from carrot and broad bean (Yokota *et al*. 1989), the *S. speciosa* ITS1–5.8S–ITS2 region was determined to be 622 bp long, and the lengths of the two spacers were 236 bp (ITS1) and 222 bp (ITS2). The lengths of the nrITS regions reported here for *Sinningia* are comparable to those from many other species of angiosperms (Baldwin *et al*. 1995; Vander Stappen *et al*. 1998; Vargas *et al*. 1999; Schnabel *et al*. 2003), including sequences from both New and Old World Gesneriaceae (Möller and Cronk 1997; Zimmer *et al*. 2002; Hughes *et al*. 2005; Roalson *et al*. 2005). The 5.8S rRNA gene was 164 bp in length in all accessions of *Sinningia* examined, which is within the range of values (160–165 bp) published for other angiosperms (Baldwin, 1993; Baldwin *et al*. 1995; Vander Stappen *et al*. 1998; Després *et al* 2003; Pelser *et al*. 2003; Schnabel *et al*. 2003). The conserved 21 bp sequence 5′-GGCGCGGCAAGCGCCAAGGAA-3′, possibly important in rRNA processing (Liu and Schardl 1994), was present in ITS1 at positions 140–160. The aligned data matrix for all full-length nrITS sequences was 624 bp, which included 1 bp gaps in ITS1 and ITS2 at positions 93 and 447, respectively. The individual alignments were 237 bp (ITS1) and 223 bp (ITS2) in length. There were 33 (5.3 %) parsimony-informative sites in the alignment, 16 in ITS1, 14 in ITS2 and three in the 5.8S RNA gene. Of the 52 singleton sites, 18 were in ITS1, one was in the 5.8S gene and 33 were in ITS2. Eighty-six per cent (537/624) of the sites were conserved. There was no missing data in the alignment.

**Table 3:** **Features of the nrITS region from *Sinningia*.** Internal transcribed spacer-specific fragments were amplified from genomic DNA isolated from 24 accessions of *S. speciosa* and one each of *S. guttata* and *S. macrophylla* using the primers ITS6 and ITS8m. The lengths of ITS1 and ITS2 were determined by comparison with sequences from carrot and broad bean (Yokota *et al*. 1989). Sequence analysis was performed with DnaSP v5 (Librado and Rozas 2009). RNA secondary structure minimum free energies (Δ*G*) were calculated individually for ITS1 and ITS2 from all nrITS sequences using the Mfold web server (**http://mfold.rna.albany.edu/**) (Zuker 2003).

nrITS sequence	
Overall length	622–623 bp
Aligned length	624 bp (2 gaps)
5.8S rDNA length	164 bp
Mean G + C content	53 %
No. of variable sites	85
Parsimony informative sites	33
Maximum sequence divergence (no./%) (all *Sinningia*)	49/7.9 (*S. guttata*—‘Carangola’)
Maximum sequence divergence (no./%) (wild *S. speciosa* only)	25/4.0 (‘Carangola’—‘Espirito Santo’)
Maximum sequence divergence (no./%) (all *S. speciosa*)	27/4.3 (‘KW-C’—‘Espirito Santo’)
Minimum free energy (Δ*G*) kcal mol^−1^—ITS1	−78.30 to −90.60 (mean = −86.98)
Minimum free energy (Δ*G*) kcal mol^−1^—ITS2	−75.90 to −90.50 (mean = −81.40)

The highest level of sequence divergence for the 27 nrITS sequences was 7.9 % between *S. speciosa* ‘Carangola’ and *S. guttata*. Within the group of 17 wild *S. speciosa* collections, divergence ranged from 0 to 4 % (Table 3), which is comparable to levels of intraspecific nrITS divergence previously reported in angiosperms, e.g. 3.7 and 1.2–1.8 %, respectively, between subspecies of *Calycadenia truncata* (Baldwin 1993) and *Caulanthus amplexicaulis* (Pepper and Norwood 2001), 0–5.2 % between members of the *Streptanthus glandulosus* complex (Mayer and Soltis 1999), 0.4–4.9 % within several populations of the Arctic endemic *Saxifraga cernua* (Brochmann *et al*. 1998) and an average of 2.4 % between European and North African populations of *Saxifraga globulifera* separated by the Straits of Gibraltar (Vargas *et al*. 1999). Higher levels of intraspecific nrITS divergence, up to 7.68 % for ITS1 + ITS2, have been reported for species of *Aeschynanthus* (Gesneriaceae; Denduangboripant *et al*. 2001), and could very well result from reduced rates of concerted evolution at the ribosomal repeat loci (Denduangboripant and Cronk 2000).

**Fig. 6:**
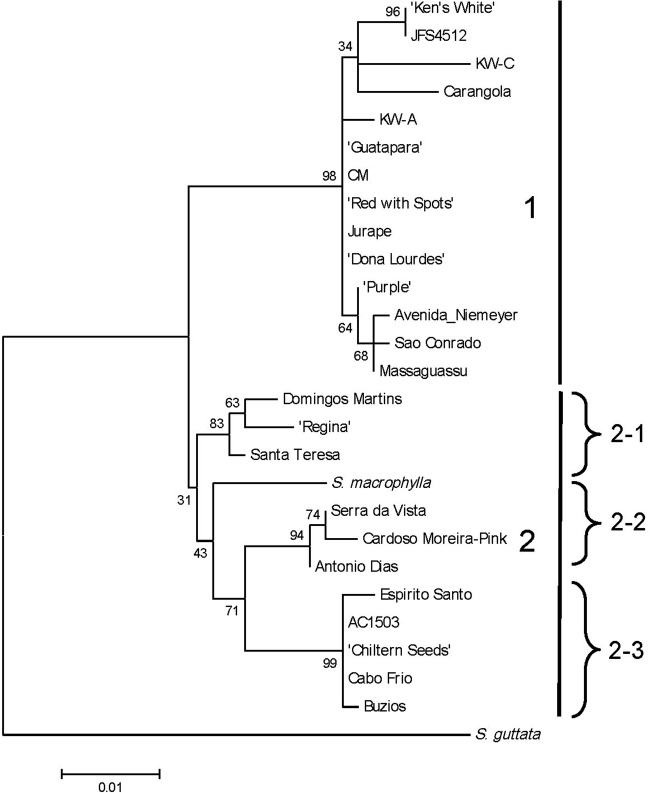
**Maximum likelihood tree calculated from the *Sinningia* nrITS DNA sequence alignment using MEGA5**. The tree with the highest log-likelihood (−ln *L* = 1482.93) is shown. Bootstrap percentages (1000 replicates) are shown adjacent to the nodes for all clades. All amplified nrITS fragments were sequenced directly with the exception of *S. speciosa* ‘Kaiser Wilhelm', where the PCR products were cloned into pGEM-T (Promega, Madison, WI). The sequences of two representative clones, KW-A and KW-C, were included in the ML analysis.

Maximum likelihood analysis with BS was performed on the *Sinningia* nrITS alignment. The resulting unrooted tree with nodal BS values is shown in Fig. 6. Maximum likelihood separated the 27 ITS sequences into two major clades, with *S. guttata* as outgroup. Clade #1, which contained all of the cultivated forms and six wild collections, had strong statistical support (98 % BS), as did the three smaller subclades 2-1, 2-2 and 2-3 (BS values ≥ 80 %). The best support was for the small clade 2-3 (99 % BS), which was nearly identical to cluster A1-1 from the Dice/UPGMA phenogram.

## Discussion

### Cluster analysis of AFLP data

A Dice/UPGMA cluster analysis was performed on the *Sinningia* AFLP data matrix to uncover possible genetic structure within a group of 24 accessions of *S. speciosa*. Included as outgroups were one accession each of *S. guttata* and *S. macrophylla*, which are phylogenetically close to *S. speciosa* (Perret *et al*. 2003) and are the only species with which it will form fertile hybrids. Phenetic cluster analysis groups individuals or species based on presently existing shared characters, which in this case are genomic DNA fragments. Phenetics is distinguished from cladistics, which seeks to reconstruct evolutionary relationships from DNA sequence alignments.

Amplified fragment length polymorphism markers are anonymous DNA fragments that are amplified from loci distributed randomly throughout the genome. The individual fragments—here detected as fluorescent peaks—are coded as binary markers (1 = present, 0 = absent) and scored as independent unit characters of equal weight. The presence of the null allele (0) makes it impossible to distinguish between the allelic states 1/1 and 1/0 in most cases, and thus AFLPs mimic dominant morphological genetic markers. Amplified fragment length polymorphism fragments are amplified under very selective conditions—each fragment is flanked by two restriction enzyme recognition sites (EcoRI and MseI in this case), and the amplification specificity is determined by the six selective nucleotides (3 + 3) internal to the restriction sites that extend beyond the 3′-end of each primer. Therefore, while a single set of criteria results in successful amplification of any given fragment, amplification failure (fragment absence) can be attributed to at least 18 distinct events: a change (mutation) in one or more of the 16 critical nucleotides (6 for EcoRI + 4 for MseI + 6 selective), or an insertion or deletion event (indel) that encompasses either of the terminal restriction sites or the sequence between them. Subsequent analyses could potentially be confounded because it is impossible to distinguish between any of the multiple events that lead to fragment absence. Dice's similarity coefficient, which is equivalent to 1 minus Nei and Li's GD (Nei and Li 1979), and Jaccard's coefficient are well suited for the analysis of AFLP data because both only consider shared characters (scores of 1) within each fragment bin, and give no weight to the shared absence of a band (including shared band absence would introduce homoplasy into the data set for the reasons given above). These methods are advantageous because band absences (scores of 0) are excluded from the analyses, and there are also no assumptions of the Hardy–Weinberg equilibrium. Phenetic analysis is therefore appropriate for use with AFLP data (Koopman *et al*. 2001; Spooner *et al*. 2005).

Scoring of non-homologous fragments (co-migrating fragments that have different sequences) is one potential source of homoplasy that can introduce error into distance estimates calculated from AFLP data (Després *et al*. 2003; Meudt and Clarke 2007). However, the few studies that have addressed this issue through DNA sequencing found a high degree of identity in the sequenced AFLP fragments (Simmons *et al*. 2007). At lower taxonomic levels, AFLP fragment-size homoplasy will be minimal, and can be expected to have little effect in intraspecific studies (Tremetsberger *et al*. 2006). Indeed, recent computer simulations based on sequenced genomes indicated that homologies of co-migrating AFLP fragments are greater between closely related taxa, and that fragment-size homoplasy is much lower in organisms with small (<400 Mbp) versus large (>2 Gbp) genomes (Althoff *et al*. 2007).

The intent of this study was to examine intraspecific relationships within a group of wild collections and cultivars of *S. speciosa*. In the Dice/UPGMA dendrogram (Fig. 3), the *S. speciosa* accessions were divided into two major clusters (A and B). Within cluster A, there was a major division that essentially separated all of the cultivars from the majority of the wild collections (secondary clusters A1 and A2). These two subclusters were further subdivided into two (A1) and three (A2) well-supported tertiary clusters. Cluster A1-1 contained three plants collected in the Cabo Frio region of south-eastern Rio de Janeiro state (AC1503, ‘Buzios’ and ‘Cabo Frio’) and had very high statistical support. The very close identity with ‘Cabo Frio’ implies that the commercial accession ‘Chiltern Seeds’ also originated in that area. The four members of cluster A1-1, including ‘Espirito Santo’, are united by their common morphology in that they are relatively small plants with distinctive bright green foliage and small lavender flowers. Cluster A1-2, which is a sister to A1-1, contains three accessions (‘Domingos Martins’, ‘Regina’ and ‘Santa Teresa’) that have dark-coloured leaves with silver veins and purple flowers. ‘Regina’ is of unknown origin (Sprague 1904), while both ‘Domingos Martins’ and ‘Santa Teresa’ originated in Espírito Santo state. ‘Carangola’, a unique form with bi-coloured flowers from Minas Gerais, also resides in cluster A1-2, although the BS support for this relationship is only 61 %. Cluster A2 contained both wild and cultivated accessions of *S. speciosa*. The four wild collections in A2 all grouped together in subcluster A2-1; ‘Avenida Niemeyer’ and ‘São Conrado’ were both collected close to the beach in the southern part of Rio de Janeiro, and are larger plants with large, dark purple flowers. ‘Jurapê’ is an unconfirmed collection from Santa Catarina state that showed affinity with ‘CM’, although the BS support (51 %) was relatively weak. Considering that the distance between Rio de Janeiro and the closest point in north-eastern Santa Catarina state (Joinville) is >900 km, the purported geographical origin of this collection could be in doubt. ‘Kaiser Wilhelm’, a peloric ‘gloxinia’ cultivar from the late 19th century, was the single cultivated accession in cluster A2-1. Clusters A2-2 and A2-3 contained only cultivated accessions, and it is interesting that the three cultivars with nodding (wild-type) flowers, ‘Dona Lourdes’, ‘Purple’ and ‘Guatapara’, grouped together in cluster A2-2 (BS = 99 %). The three remaining cultivars in cluster A (JFS4512, ‘Ken's White’ and ‘Red with Spots’) all have peloric flowers and formed a well-supported subcluster (A2-3; BS = 85 %). Cluster B (BS = 99 %) contained *S. macrophylla* and three wild collections of *S. speciosa*—‘Antônio Dias’, ‘Cardoso Moreira-pink’ and ‘Massaguassu’. ‘Antônio Dias’ and ‘Cardoso Moreira-pink’ have elongated stems and are morphologically distinct from the other wild forms of *S. speciosa* included in this study.

Principal coordinates analysis was also used to visualize the relationships within the group of 26 *Sinningia* accessions. Principal coordinates analysis is an ordination method similar to the principal components analysis, and both are frequently applied to the exploration of AFLP data (Koopman *et al*. 2001; Kim *et al*. 2004; Chao *et al*. 2005; Fernandez *et al*. 2006; Azizi *et al*. 2009). Cluster assignments from the *Sinningia* Dice/UPGMA tree (Fig. 3) were preserved in the PCoA (Fig. 4), and they supported the results of the AFLP phenetic analysis. Two minor differences were noted between the two analyses: (i) in the PCoA, AC1503 is somewhat removed from the other members of cluster A1-1 (‘Buzios’, ‘Cabo Frio’, ‘Chiltern Seeds’ and ‘Espirito Santo’), and (ii) ‘Serra da Vista’ is included in PCoA cluster A1-2, whereas it was placed between the major clusters A and B in the Dice/UPGMA tree. Principal coordinates analysis also shows that the plants in cluster B do not appear to be closely affiliated with the other *S. speciosa* accessions.

### Phylogenetic analysis of the nrITS region in *S. speciosa*

A phylogenetic analysis of the nrITS region was also performed for *S. speciosa*. The nrITS sequence is a popular tool in plant systematics, and has been used for investigating species relationships in both Old World and New World Gesneriaceae (Möller and Cronk 1997; Zimmer *et al*. 2002; Clark and Zimmer 2003; Roalson *et al*. 2005; Clark *et al*. 2009; Wang *et al*. 2011). The nrITS region is most informative at higher taxonomic levels (species and above), although intraspecific ITS sequence variation has been documented in several angiosperm dicot families such as the Apiaceae (Vargas *et al*. 1998), Asteraceae (Baldwin 1993), Brassicaceae (Mayer and Soltis 1999; Pepper and Norwood 2001), Fabaceae (Vander Stappen *et al*. 1998), Saxifragaceae (Brochmann *et al*. 1998; Vargas *et al*. 1999), Lamiaceae (Prather *et al*. 2002) and Gesneriaceae (Hughes *et al*. 2005). In the present study, the nrITS region (ITS1–5.8S–ITS2) was amplified from the same 26 *Sinningia* accessions included in the AFLP analysis. Unambiguous nucleotide sequences were obtained for 25 accessions directly from the purified PCR products. Repeated sequencing failures necessitated cloning of the amplified fragments from ‘Kaiser Wilhelm’, however. Sequencing of several recombinant plasmids revealed that the ITS region is polymorphic in this particular cultivar, and sequences from two representative clones were included in the alignment. Intra-individual nrITS sequence polymorphism can result from incomplete concerted evolution, the process by which repetitive sequences in a gene family become homogenized through gene conversion (Liao 2008). Incomplete concerted evolution of the rRNA gene repeats is suggested in the ancient gymnosperm *Cycas*, due to the presence of highly polymorphic functional nrITS repeats as well as many divergent ITS paralogues isolated from individual plants (Xiao *et al*. 2010). Incomplete concerted evolution is also apparent in taxa of hybrid origin in Rosaceae (Campbell *et al*. 1997; Ritz *et al*. 2005), and in many species of *Aeschynanthus*, a genus of south-east Asian Gesneriaceae (Denduangboripant and Cronk 2000).

The ML tree (Fig. 6) shows that the nrITS DNA sequence is phylogenetically informative in *S. speciosa*, although several nodes had BS support of <50 %. The most noteworthy feature of the phylogram is that many of the clusters present in the AFLP analyses are retained in the ML tree. Significantly, all of the ‘gloxinia’ cultivars—regardless of flower form—grouped together in one large, well-supported clade (clade #1) along with the two known collections from Rio de Janeiro state (‘Avenida Niemeyer’ and ‘São Conrado’). The two ITS sequences from ‘Kaiser Wilhelm’ reside in this clade, even though they differ at five sites each in ITS1 and ITS2 (nine transitions and one transversion). There is also a 1 bp insertion (T) in KW-C ITS1, which probably accounted for the difficulty sequencing the mixed ITS amplicons directly. Pairings between ‘Avenida Niemeyer’ and ‘São Conrado’ and between ‘Ken's White’ and JFS4512 are retained from the AFLP tree, and clade #1 is nearly equivalent to Dice/UPGMA cluster A2. Major differences are the inclusion of ‘Carangola’ and ‘Massaguassu’ in this clade. Also retained in the ML tree is the ‘Antônio Dias’–‘Cardoso Moreira-pink’ pairing in clade 2-2, and the close relationship between the three collections with dark leaves and silver veins (‘Regina’, ‘Domingos Martins’ and ‘Santa Teresa’) in clade 2-1.

## Conclusions and forward look

Independent phenetic and phylogenetic analyses of two different data sets (AFLPs and nrITS DNA sequences, respectively) show that the available wild collections of *S. speciosa* comprise a genetically diverse group; estimates of GD calculated from the AFLP data are positively correlated with geographic distance, indicating that the observed diversity could result from reproductive isolation. Unfortunately, there has been no detailed population survey conducted for *S. speciosa*, and its present geographical range is poorly delimited at best. In addition, some of the wild collections included here (e.g. ‘Jurapê’ and ‘Massaguassu’) came with anecdotal collection information. For these reasons, this study must be considered preliminary. Nevertheless, several conclusions can be drawn from the results presented here. (i) All of the ‘gloxinia’ cultivars reside in cluster A2 and clade #1, evidence that the primary gene pool for the cultivars probably came from plants originating in Rio de Janeiro. This makes sense, because historical records state that several of the early ‘gloxinias’ exported to Europe were collected within or near this city (Hooker 1842; Paxton 1846; Brackenridge 1886). However, the separation of three of the erect-flowered cultivars in cluster A2-3 (Fig. 3) could be an indication that plants from at least one other geographic region contributed genetically to the cultivars grown today. (ii) Plants collected in the Eastern Cape region of Rio de Janeiro state and very southern Espírito Santo state form a cohesive group. These plants are distinct from those found ∼160 km to the west of the city of Rio de Janeiro and also from plants collected in eastern Minas Gerais and north-eastern Espírito Santo states. (iii) The unknown wild collection ‘Regina’ groups with morphologically similar plants having dark leaves with silver veins in phenetic cluster A1-2 and phylogenetic clade #2-1, and was therefore most probably collected in Espírito Santo state. (iv) The three cultivars with wild-type (nodding) flowers (‘Dona Lourdes’, ‘Guatapara’ and ‘Purple’) in AFLP subcluster A2-2 are distinct from the other cultivars with peloric (erect) flowers. ‘Dona Lourdes’ and ‘Guatapara’ have much larger nuclear genomes than any other accessions of *S. speciosa* (Zaitlin and Pierce 2010) and appear to be tetraploids, which could be one factor to account for the observed separation from the other cultivars. (v) The wild collection ‘Jurapê’ is almost certainly not from Santa Catarina state, since both analyses group it with plants from Rio de Janeiro and the cultivars. (vi) The taxonomic position of *S. macrophylla* may need to be re-examined. A previous phylogenetic treatment using combined sequence data from both chloroplast and nuclear DNA placed *S. macrophylla* as the sister species to *S. speciosa* (Perret *et al*. 2003). The two species share many morphological characters, but the genome of *S. macrophylla* is ∼25 % larger than that of *S. speciosa* (Zaitlin and Pierce 2010). In addition, *S. macrophylla* has a very restricted range in the dry forests of southern Bahia, well isolated from known populations of *S. speciosa* (Thomas 2007; Araujo and Chautems 2010). In the present study, the phenetic analysis places *S. macrophylla* in AFLP cluster B with several divergent forms of *S. speciosa* (Figs 3 and 4), and it is nested within *S. speciosa* in clade #2 in the ML tree (Fig. 6). Further work using molecular markers (see below) and/or other DNA sequences will be required to clarify the relationship between these two taxa.

*Sinningia speciosa* is not listed in the IUCN Red List of Threatened Species (**http://www.iucnredlist.org**), although many other unlisted species of *Sinningia* meet the criteria for inclusion in the endangered or critically endangered IUCN categories (Chautems *et al*. 2010). *Sinningia speciosa* is distributed over a relatively large area, but has almost certainly experienced considerable habitat disruption given that the Mata Atlântica presently exists in ∼245 000 forest fragments, many of which are small, <100 ha in size (Ribeiro *et al*. 2009). Recent observations of *S. speciosa* in the field in Brazil (D.Z., December 2011) revealed that (i) populations are often isolated and may consist of very few individuals; (ii) populations do not overlap (they are not sympatric); (iii) many populations grow outside of protected areas, sometimes adjacent to agricultural enterprises; and (iv) the various diverse morphological forms are locally endemic, and some are quite rare. For these reasons, there is an urgent need to discover, describe and systematically sample wild populations of *S. speciosa* for genetic diversity. This laboratory is presently developing a suite of co-dominant genetic markers based on simple sequence repeats (Morgante *et al*. 2002) and intron sequences from annotated genes. These markers are highly informative, and will be invaluable for genotyping wild and cultivated germplasm, and for evolutionary studies in *S. speciosa* and its relatives. More importantly, we will be able to quantify the levels of allelic diversity and the extent of gene flow within and between populations of *S. speciosa*. This in turn will allow us to define evolutionarily significant units (Moritz 2002), and make recommendations for conservation of this interesting and unique species.

## Additional information

The following additional information is available in the online version of this article –

File 1: Large population of *S. speciosa* growing on a granite wall along the coastal road Avenida Niemeyer in the southern part of Rio de Janeiro, Brazil. Photograph taken on 27 November 2011 by D. Zaitlin.

File 2: A population of *S. speciosa* growing on a slope adjacent to the sea near the town of Armação dos Búzios, RJ, Brazil. Photograph taken on 28 November 2011 by D. Zaitlin.

File 3: A dark-leaved form of *S. speciosa* growing on a moist bank above a dirt road near Córrego da Xica, RJ, Brazil. Photograph taken on 28 November 2011 by D. Zaitlin.

File 4: A population of a dark-leaved form of *S. speciosa* with bi-coloured flowers growing adjacent to a stream near the Hotel Fazenda Pedra Lisa, RJ, Brazil. Photograph taken on 29 November 2011 by D. Zaitlin.

File 5: A caulescent form of *S. speciosa* growing at the top of a hill with cacti and spikemosses near the town of Cardoso Moreira, RJ, Brazil. Photograph taken on 29 November 2011 by D. Zaitlin.

## Accession numbers

DNA sequences of the *Sinningia* nrITS region (ITS1 + 5.8S RNA gene + ITS2) used in this study have been deposited with GenBank. Accession numbers are: JQ928140 (*S. macrophylla*), JQ928141 (*S. guttata*) and JQ928142 to JQ928165 (wild and cultivated *S. speciosa*).

## Sources of funding

The author acknowledges Kentucky Tobacco Research and Development Center for providing laboratory space and scientific infrastructure, and Dr H.M. Davies and the Kentucky Tobacco Research Board for supporting this research.

## Conflict of interest statement

None declared.
